# The MTT Assay: Utility, Limitations, Pitfalls, and Interpretation in Bulk and Single-Cell Analysis

**DOI:** 10.3390/ijms222312827

**Published:** 2021-11-26

**Authors:** Mahshid Ghasemi, Tyron Turnbull, Sonia Sebastian, Ivan Kempson

**Affiliations:** Future Industries Institute, University of South Australia, Adelaide, SA 5095, Australia; mahshid.ghasemi_esfidvajani@mymail.unisa.edu.au (M.G.); Tyron.Turnbull@unisa.edu.au (T.T.); sonia.sebastian@mymail.unisa.edu.au (S.S.)

**Keywords:** MTT assay, how to, interpret, cell metabolism, viability assay, cytotoxicity, gold nanoparticles, PC-3 cells, image cytometry

## Abstract

The MTT assay for cellular metabolic activity is almost ubiquitous to studies of cell toxicity; however, it is commonly applied and interpreted erroneously. We investigated the applicability and limitations of the MTT assay in representing treatment toxicity, cell viability, and metabolic activity. We evaluated the effect of potential confounding variables on the MTT assay measurements on a prostate cancer cell line (PC-3) including cell seeding number, MTT concentration, MTT incubation time, serum starvation, cell culture media composition, released intracellular contents (cell lysate and secretome), and extrusion of formazan to the extracellular space. We also assessed the confounding effect of polyethylene glycol (PEG)-coated gold nanoparticles (Au-NPs) as a tested treatment in PC-3 cells on the assay measurements. We additionally evaluated the applicability of microscopic image cytometry as a tool for measuring intracellular MTT reduction at the single-cell level. Our findings show that the assay measurements are a result of a complicated process dependant on many of the above-mentioned factors, and therefore, optimization of the assay and rational interpretation of the data is necessary to prevent misleading conclusions on variables such as cell viability, treatment toxicity, and/or cell metabolism. We conclude, with recommendations on how to apply the assay and a perspective on where the utility of the assay is a powerful tool, but likewise where it has limitations.

## 1. Introduction

The MTT reagent (3-(4,5-dimethylthiazol-2-yl)-2,5-diphenyl-2H-tetrazolium bromide) is a mono-tetrazolium salt that consists of a positively charged quaternary tetrazole ring core containing four nitrogen atoms surrounded by three aromatic rings including two phenyl moieties and one thiazolyl ring. Reduction of MTT results in disruption of the core tetrazole ring and the formation of a violet-blue water-insoluble molecule called formazan [1]. The MTT reagent can pass through the cell membrane as well as the mitochondrial inner membrane of viable cells presumably due to its positive charge [1] as well as its lipophilic structure [2] and is reduced to formazan by metabolically active cells [1]. The chromogenic nature of this redox chemical reaction provides a colorimetric-based measurement of intracellular formazan production based on which the MTT assay was developed by Mosmann et al. in 1983 [3]. Consequently, the assay has extensive utility as a cell metabolic activity assay. However, its utility has increasingly been applied to infer secondary processes or states of cells, such as viability, which is frequently unsubstantiated.

The MTT assay is typically performed after a few hours of incubation of cells with MTT. The water-insoluble formazan produced is then solubilized by a solvent such as Dimethyl sulfoxide (DMSO). Subsequently, the lowering of the light transmission by absorbance and other mechanisms by the homogenized MTT-formazan solution is measured by a microplate reader in terms of its optical density (OD) at a wavelength which MTT-derived formazan absorbs the most (around 570 nm). The measured OD values are assumed to be a representation of formazan concentration and consequently the intracellular reduction of MTT. This has been the basis of applying the MTT assay for nearly four decades as a common tool to measure cell proliferation/viability, drug cytotoxicity, and mitochondrial/metabolic activity of cells [2].

Intracellular reduction of MTT can be mediated by oxidoreductase and dehydrogenase enzymes and electron donors (mainly NAD(P)H) at different stages of the glycolytic pathways to the mitochondrial electron transport chain [1]. The location of formazan formation and its intracellular transportation has remained controversial. While the role of mitochondria in MTT reduction [2,4,5] has been a justification for the common application of the assay to measure mitochondrial activity [6,7,8,9,10,11,12], biochemical and microscopic studies have located formazan in various intracellular organelles. Intracellular formazan granules have been observed in the endoplasmic reticulum, cytosolic lipid droplets [13,14], plasma membranes [1,15], nucleus, and microsomes [5]. These observations suggest that the MTT assay is more than a mere representation of mitochondrial activity [2]. Furthermore, there are several biomolecules such as ascorbic acid, cysteine, dihydrolipoic acid, glutathione, glutathione S-transferase, and tocopherols that can also reduce MTT [2].

Several studies have revealed limitations of the MTT assay [2,3,4,5,6,7,8] and various confounding factors that are needed to be considered in designing, performing, analyzing, and interpreting the assay results [5,9,10,11,12,13,14]. However, the MTT assay is still commonly used and interpreted, overlooking these limitations and escaping the necessary optimization steps. Commonly overlooked confounding variables include seeding cell number, the concentration of MTT reagent added to the cells, time of incubating cells with MTT, type of culture media, cells’ supernatant removal following MTT incubation, the wavelength at which optical density is measured, and the tested treatment. Inconsistencies also exist in these parameters which make it difficult to compare the measured OD values between different studies. Moreover, the common purporting as a viability assay is often erroneous. Reduction of the dye depends primarily on cell metabolism; sometimes this is reflective of cell viability, but confounding variables means this often leads to the inaccurate utility of the assay.

The underlying mechanism of the assay has also not been fully understood and there are still controversies and uncertainties on some of the aspects such as what additional organelles, enzymes, and molecules are involved in MTT reduction, the origin of extracellular formazan crystals, the cytotoxic effect of the MTT reagent itself, and how the assay measurements represent cell viability, metabolic activity, and/or treatment toxicity [2]. 

In this report, with the aim of assessing the applicability and limitations of the MTT assay, we apply the assay in a typical context of testing polyethylene glycol (PEG)-coated gold nanoparticle (Au-NP) toxicity in a prostate cancer cell line (PC-3). This is representative of how the assay is very commonly applied to test ‘cell viability’ in the literature. However, we thoroughly assessed the effect of cell seeding density, MTT concentration, MTT incubation time, and serum starvation on the measured values. We also investigated the potential confounding effects of various factors on the assay measurements including cell culture media components such as phenol red, released intracellular contents (cell lysate and secretome), extrusion of formazan to extracellular space, and the PEG-coated Au-NPs (PEG-Au-NPs) themselves. In this context, we present our data in hand with a critical assessment of the literature to assess and discuss the applicability and potential limitations of the assay. We additionally test and discuss the limitations of microscopic image cytometry as a tool for measuring intracellular MTT reduction at the single-cell level.

**Figure 1:**
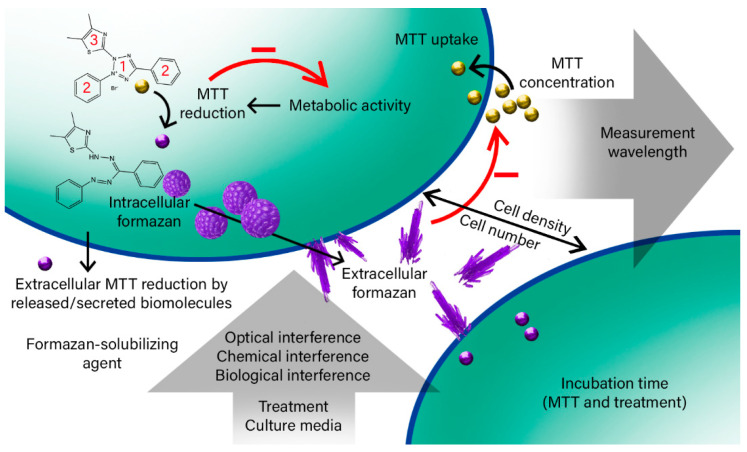
Factors affecting the final optical density (OD) measurements in the MTT assay. These include the concentration of MTT reagent and the proportion that actually enters the cell, cellular metabolic activity (which is highly dependent on a multitude of variables including treatments to the cells, biological effect of culture media, cell density, and impedance of cell metabolism due to toxic effects of MTT), cell number, timing of formazan crystals extrusion (which could impede further MTT uptake), chemical interference such as abiotic reduction of MTT by culture media, the tested treatment, or released cellular content, optical interference by all the background components, time of incubating cells with MTT reagent and/or tested treatment, and ultimately the optical measurement. Chemical structure of MTT and formazan are illustrated inside the cell: MTT consists of a tetrazole ring core containing four nitrogen atoms (1) surrounded by three aromatic rings including two phenyl moieties (2) and one thiazolyl ring (3). Reduction of MTT results in disruption of the core tetrazole ring and the formation of formazan. Red arrows and the “-“ sign indicate disruption of MTT reduction on the normal metabolic activity of the cells and the impeding effect of the formazan crystals (when presenting on the cell surface) on further uptake of MTT reagent by cells.

Based on our findings, the assay in itself is relatively straightforward and benefits from ease of its utility. However, this utility should not be flippantly applied as a simple cell viability test as is all too commonly prevalent in the literature. Rather, a rigorous process should be undertaken to confirm optimization of the assay followed by a rational interpretation of the data keeping in mind what the assay actually represents, i.e., being a measurement of how much reduced reagent exists in the sample. This depends on the number of cells, the amount of reagent that actually enters the cell, cellular metabolic activity (which is highly dependent on a multitude of variables including treatments to the cells), the timing of formazan crystals extrusion, the cytotoxic effect of MTT, abiotic reduction of MTT by culture media or the tested treatment, and ultimately the optical measurement which may also experience interference from the culture media or the tested treatment such as nanoparticles (Figure 1). We conclude with recommendations on how to apply the assay and a perspective on where the utility of the assay is a powerful tool, but likewise where it has limitations.

## 2. Results and Discussion

### 2.1. The Effective Factors on the Bulk MTT Assay Measurements

To define the variables that could potentially affect the bulk MTT assay measurements, we assessed optical density (OD) (specifically, the reduced transmission intensity of light when the sample is being illuminated with a bandwidth of 10 nm from 565 to 575 nm, denoted as 570/10 nm) as a function of cell seeding number, MTT concentration, and the time of incubating cells with MTT reagent. Furthermore, we assessed if the released intracellular contents as a result of cell lysis or secretion contribute to MTT reduction. We also investigated how the culture media components, the extracellular formazan, and the Au-PEG NPs may contribute to the measured OD.

#### 2.1.1. Cell Number and Density

**Figure 2:**
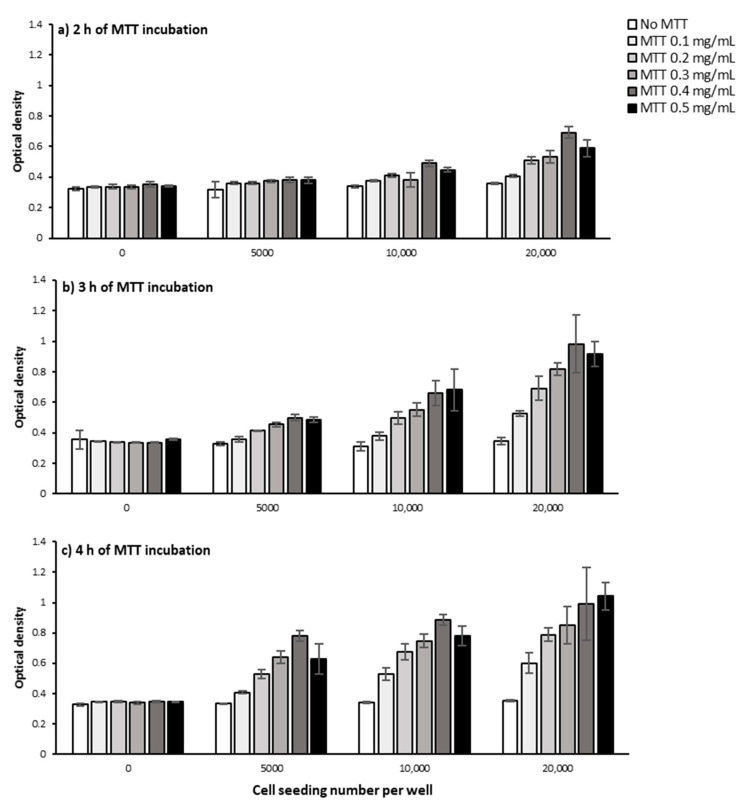
OD depends on cell seeding number/density, MTT concentration, and incubation time: OD changes with increasing cell seeding number/density. PC-3 cells were allowed to grow for 26 h before MTT addition. Absorbance was measured following 2 h (**a**), 3 h (**b**), and 4 h (**c**) of incubating cells with different concentrations of MTT. Data shown as mean OD of triplicate wells and error bars represent standard deviation (SD).

**Figure 3:**
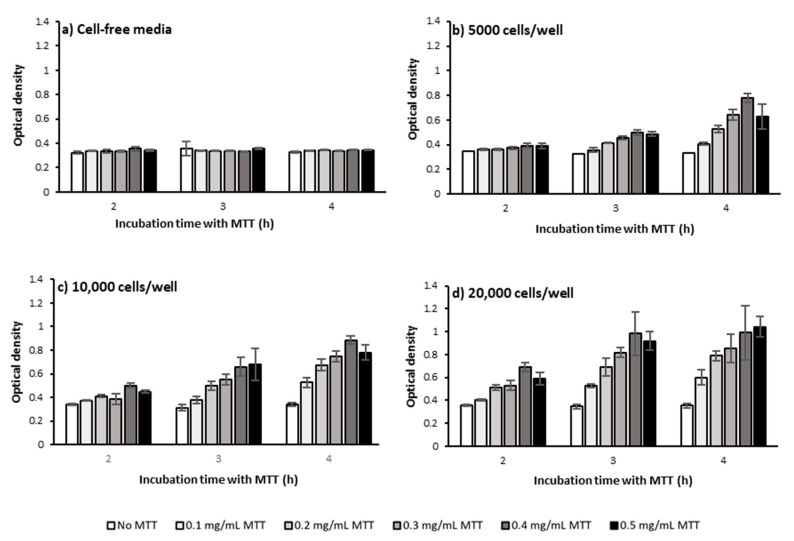
OD depends on cell seeding number/density, MTT concentration, and incubation time: OD changes with increasing MTT incubation time. (**a**) OD of cell-free culture media (Phenol red-containing RPMI + 10% FCS) at different time points after incubation with different concentrations of MTT. (**b**–**d**) OD of PC-3 cells with different seeding numbers of 5000 (**b**), 10,000 (**c**), and 20,000 (**d**) per well of 96-well plates. Cells were allowed to grow for 26 h before the addition of different concentrations of MTT and the OD was measured at different time points following MTT addition. Data shown as mean OD of triplicate wells and error bars represent SD (standard deviation).

**Figure 4:**
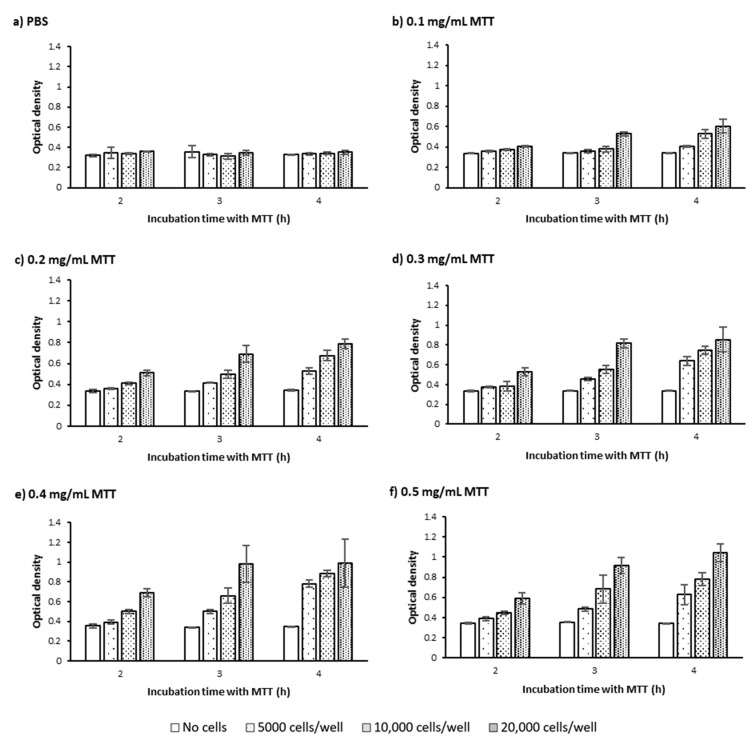
OD depends on cell seeding number/density, MTT concentration, and incubation time: OD changes with increasing MTT incubation time. PC-3 cells were allowed to grow in 96-well plates for 26 h before incubation with MTT. OD was measured at different time points after addition of 10 µL of (**a**) PBS, or (**b**) 0.1 mg/mL, (**c**) 0.2 mg/mL, (**d**) 0.3 mg/mL, (**e**) 0.4 mg/mL, and (**f**) 0.5 mg/mL of MTT–PBS solutions to 100 µL of each well content. Data shown as mean OD of triplicate wells and error bars represent standard deviation (SD).

Figure 2 presents OD as a function of cell seeding number and how it varies for varying concentrations of MTT and incubation time. As the data indicates, regardless of MTT concentration and MTT incubation time, increasing cell seeding number increases OD (Figure 2). This is intuitive as increasing the number of cells accordingly increases the total level of produced formazan by the cell population and consequently the measured OD, as shown in other studies [16,17,18]. The fact that the OD levels in the absence of MTT did not change with increasing the cell number shows this is not due to the optical effects of increased cell number (Figure 3 and Figure 4a).

The observed increase in OD could also be a result of an increase in cell density, rather than the number of cells per se, given that spatial proximity of the cells to each other can affect intercellular communication and consequently alter the metabolic behavior of the cells [19,20,21]. In other words, not only does the sum of formazan levels produced by cells increase as the number of cells rises, but also higher cell density may also change the level of produced formazan in every single cell through altering the nature of intercellular signaling and consequently the cells’ enzymatic activity. This is not however merely drawable from our data and needs to be confirmed by comparing the OD levels of different cell populations with the same seeding number in vessels of different sizes.

Reaching a plateau level in OD beyond a certain number of cells has also been reported previously [16], attributed to the over confluence caused by seeding high numbers of cells and consequent changes in nutrient availability and cell viability and metabolism [16,22]. The fact that we did not observe a saturation level in OD by increasing cell number, is not an unexpected finding given the maximum seeding number used in this work (20,000 cells/well of Corning^®^ 96-well Microplate, area per well: 0.32 cm^2^) is expected to correspond to approximately 90% confluence at the time of the measurements (27–30 h after seeding) based on our light microscopy observations.

Having a closer look at the graphs in Figure 2, it is also notable that the effect of increasing cell number on OD is not of the same magnitude among different MTT concentrations and incubation time points. Even at a given MTT concentration and incubation time-point, the magnitude of this effect does not remain constant as the cell number increases. For instance, after 3 h of incubating cells with 0.1 mg/mL MTT (Figure 2b), doubling the seeding number from 5000 to 10,000 cells per well only causes a 5% increase in OD (from 0.357 to 0.378), while doubling from 10,000 to 20,000 cells/well causes a 39% increase (from 0.378 to 0.527) in OD. This also provides a direct indication that changes in the OD levels are not necessarily a direct measurement of changes in the percentage of viable cells, i.e., doubling the cell number does not result in a doubling of OD level. Other studies have also shown that OD is not always a linear function of cell number, and the function varies depending on cell type [16,18], cell number [16,17,18], pH [18], and the formazan-solubilizing agent [17]. Moreover, it was previously shown that a higher number of cells could cause a shift in the UV-Vis spectrum peak possibly due to changes in pH. This shift could consequently result in an underestimation of formazan production for higher cell numbers [18].

Altogether, these findings suggest that before implementing the MTT assay to assess cell viability, stringent control experiments must be performed as a means to determine how the aforementioned parameters influence the relationship between the measured OD and cell number. This is however commonly overlooked in the published studies, where OD values are used as a proxy for cell viability percentage and consequently erroneous quantitative measurements of treatment efficacy or drug-induced cytotoxicity [23,24,25,26,27,28,29].

To infer cell viability based on OD values, the assay may still suffer from unintended bias for further reasons. Formazan concentration produced by a cell population is affected by more than just the number of viable cells. For instance, other factors due to the testing treatment/condition(s) can also affect results such as differing amounts of MTT uptake [5,15] and changes in metabolic activity [30]. In other words, the net sum effect of all these factors, and not just one of them, determines the level of MTT reduction, consequent formazan production, and finally the measured OD. For instance, it is possible for two cell populations, one with a low cell number but a high metabolic activity at the single-cell level (for example due to a cytotoxic treatment such as radiation [31,32,33,34]), and the other with a higher cell number but lower metabolic activity at the single-cell level, to show the same OD levels. If the assay was applied within the context of assessing cell viability the data could be mistakenly interpreted as showing the same level of cell viability. Such an error could be minimized if the consistency of potential confounder variables between the samples is confirmed. However, due to the unknown effects of the studied treatments/conditions on each of these variables, such consistency might not always be achievable. A good example is a study published by Mirzayans et al. where they reported more than a 10-times increase in MTT reduction at the single-cell level when treating cancer cells with the half-maximal inhibitory concentration (IC50) of cisplatin (a chemotherapeutic agent), determined using a multiwell metabolic assay [35]. This was shown to be a result of a growth-arrest response induced by cisplatin through the emergence of greatly enlarged cells that can remain viable and highly metabolically active, reduce MTT, and lead to cancer relapse. On the other hand, the emergence of these cells is also indicative of the proliferation blocking effect of cisplatin on cancer cells which was underestimated using multiwell metabolic assays [35]. A similar growth arrest response was shown to be the dominant effect of radiation therapy, rather than apoptosis, on prostate cancer cells [36]. The MTT assay measurements, however, cannot be reflective of these growth-arrested cells as a type of induced “reproductive” cell death [36]. Other than high metabolic activity, these chemo/radio-induced giant cells have been shown to also express a senescence-like phenotype that does not only include proliferation arrest but also a specific secretory phenotype, called senescence-associated secretory phenotype (SASP) [37]. This secretory phenotype can promote cancer progression via stimulating the proliferation of nearby cells, and therefore increasing the number of cells [37,38]. The SASP was also shown to contain reactive oxygen species such as superoxide [13] that can potentially contribute to MTT reduction [39]. Therefore, response to a treatment can be a complex combination of treatment effects on cell viability, proliferative ability, cell secretome, and metabolic activity at the single-cell and cell population level. Consequently, even under optimized conditions with stringent controls when the MTT assay is used to measure cell viability and/or metabolic activity, the results should be, where possible, confirmed with complementary assays to attain a more comprehensive perspective of the treatment response.

#### 2.1.2. MTT Incubation Time

Measuring OD levels at different time points (2, 3, and 4 h) following incubating PC-3 cells with MTT showed that regardless of MTT concentration and cell seeding number, increasing the MTT incubation time increased OD levels which presumably represent MTT reduction and formazan concentration (Figure 3b–d and Figure 4b–f). However, in the absence of cells, the OD levels were similar at different incubation times regardless of MTT concentration (Figure 3a).

Looking more closely at the graphs in Figure 3c and Figure 4b–e, the rate of time-related increase in OD levels also generally increases. However, the increase rate in OD declines over time for the seeding number of 20,000 cells/well at all MTT concentrations (Figure 3d and Figure 4b–f) and 10,000 cells/well at the MTT concentration of 0.5 mg/mL (Figure 4c,f). The OD finally reaches a plateau level after 3 h of incubating 20,000 cells/well with 0.3 and 0.4 mg/mL, (Figure 4d,e), but not with 0.5 mg/mL of MTT (Figure 4d,f).

This result shows that the maximum possible level of MTT reduction is reached after 3 h of incubating 20,000 PC-3 cells per well of a 96-well plate with 0.3 and 0.4 mg/mL of MTT. However, at the lower levels of cell number and/or MTT concentration, the maximum (saturation) level of MTT reduction is not reached up to 4 h of MTT incubation. In other words, the saturation time point depends on the seeded cell number and MTT concentration, as previously shown [16].

Reaching a plateau level in OD after a certain time of incubating cells with MTT might be due to reduction of all the available MTT reagent by cells or an impedance in further MTT uptake when a certain amount of formazan crystals appear at the cell surface [5,13,40]. Saturation of the cells’ metabolic capacity in reducing MTT could also be a reason for this observation. Another reason could be the cytotoxic effect of MTT. Intracellular metabolism of MTT has been shown to gradually cause mitochondrial injury, disturbance of normal cell metabolism, and finally cell apoptosis [4,41]. Time-dependent loss of membrane integrity as a result of formazan exocytosis has also been proposed as a mechanism of cell death following MTT incubation [41].

The fact that the saturation in OD levels occurs earlier with higher MTT concentration and higher cell number suggests that a higher MTT concentration and/or higher number of cells potentially accelerate reaching the saturation level in MTT reduction. A higher number of cells could result in more rapid consumption of the available MTT by the whole cell population. In addition, this could be hypothetically attributed to the effect of the proximity of the cells (cell density) on the rate of formazan production at the single-cell level as discussed earlier in Section 2.1.1. In other words, the higher density of cells and their proximity to each other may have increased the metabolic activity of PC-3 cells and thus accelerated MTT reduction and formazan production at the single-cell level. Therefore, the saturation level in OD levels, through the above-mentioned mechanisms, has been reached at an earlier time point.

**Figure 5:**
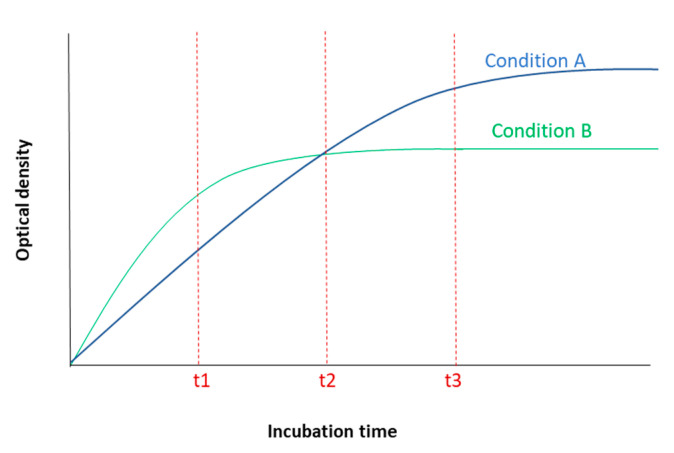
The optimum OD measurement time depends on how the OD levels change as a function of incubation time in all the tested conditions. A hypothetical graph showing OD levels as a function of MTT incubation time in two different conditions A and B. Each condition has resulted in a different saturation level and saturation time point and thus measurement time points (t1, t2, and t3 defined by dotted lines) that can affect the comparison of two conditions. Therefore, considering incubation time in comparative analysis gives us more accurate information on how each condition affects the behavior of the cell over time.

The tested treatment can also change the saturation timepoint and/or saturation level and thus can potentially confound the comparative analysis between different conditions. For instance, a treatment with enhancing effect on cell metabolism can cause the plateau level to be reached earlier compared to non-treated cells, while a metabolism-inhibitor treatment can delay the saturation time point. Therefore, OD measurement at each different time point can potentially result in different and even contradictory conclusions (Figure 5). Measuring the OD levels at more time points therefore can give us more accurate information on how each treatment affects the cells’ behavior in terms of MTT reduction as a function of time and prevents erroneous conclusions.

#### 2.1.3. MTT Concentration

**Figure 6:**
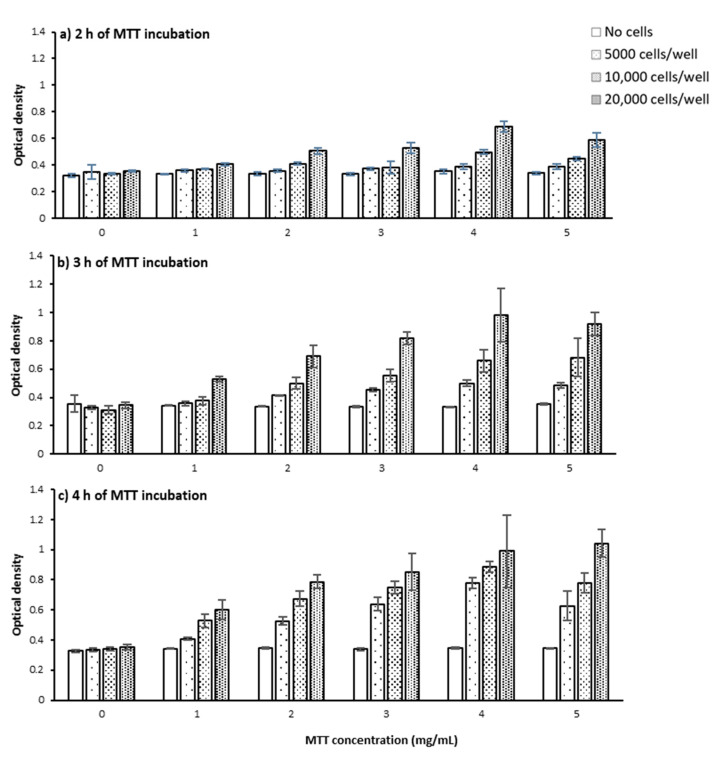
OD depends on cell seeding number/density, MTT concentration, and incubation time: OD changes with increasing MTT concentration. PC-3 cells were seeded at different numbers in 96-well plates and were allowed to grow for 26 h before adding 10 µL MTT–PBS solution of different concentrations (0–0.5 mg/mL) to 100 µL of each well content. OD was measured following 2 h (**a**), 3 h (**b**), and 4 h (**c**) of incubating cells with MTT. Data shown as mean OD of triplicate wells and error bars represent SD (standard deviation).

Our results from the MTT assay on PC-3 cells applying a range of MTT concentrations (0, 0.1, 0.2, 0.3, 0.4, 0.5 mg/mL) showed that increasing the MTT concentration up to 0.4 mg/mL caused an increase in the measured OD levels, regardless of cell number and measurement time point (Figure 6). At higher cell numbers and longer time points though, the OD difference between MTT concentrations is more obvious (Figure 6). However, increasing MTT concentration from 0.4 to 0.5 mg/mL did not result in any further increase in OD levels, regardless of cell number and measurement time point (Figure 6). A decrease [16] or plateau [18] in OD levels above a certain MTT concentration was also reported previously and shown to vary depending on the tested cell type. A likely explanation for these observations is an acceleration of MTT-induced cell death at higher MTT concentrations. In other words, above a certain level of MTT concentration, the accelerated rate of cell death is at a level that does not allow cells to reduce MTT as much as they do at lower MTT concentrations. Hence, the MTT concentration at which the maximum level of formazan is produced before MTT-induced cell death should be selected as the optimum MTT concentration for each specific cell type, cell number, and incubation time. However, as we earlier discussed the measurement time point, the tested treatment may also change the pattern of cell response to increased MTT concentration. Therefore, when the optimum MTT concentration is meant to be selected to perform a comparative analysis between different treatments, the OD levels need to be measured as a function of MTT concentration under all the conditions. Overlooking the importance of optimizing MTT concentration could result in biased measurements in the MTT assay as previously shown [16].

The main aim of our study was to assess the potential confounding effect of various factors including the tested treatment (i.e., Au-PEG nanoparticles) and not to measure the treatment toxicity. Therefore, we performed our later experiments based on the parameters that our preliminary results showed would represent the maximum capacity of each cell population in reducing MTT (cell seeding number: 20,000/well of 96-well plate, MTT incubation time: 3 h, and MTT concentration: 0.4 mg/mL). For toxicologic studies, however, these parameters need to be optimized considering the tested treatments as well as the control conditions.

#### 2.1.4. Cell Lysate and Secretome

The MTT assay is based on the intracellular reduction of MTT to purple formazan granules. Other than the intracellular granular form of formazan, microscopic studies have also reported formazan aggregates to appear as needle-shaped crystals on the cell surface or extracellularly. Some studies have proposed that these crystals are products of extracellular reduction of MTT by the culture media or the assessed treatment [42,43]; or reduction inside the plasma membrane [15,44]. An intracellular origin of these crystals has also been shown in many studies proposing their extrusion or extension into the extracellular space through perforating plasma membrane [1], exocytosis [5,40,41], or physicochemical processes at the molecular level [13]. Given the contradictory reports, however, it is not clear if formazan crystals cause plasma membrane injury [5,13,15,40,41].

**Figure 7:**
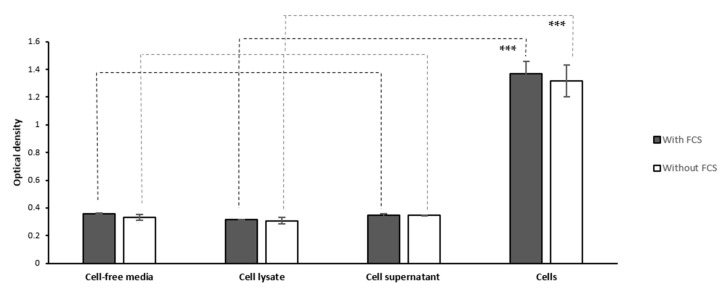
Intact, viable cells were necessary for MTT reduction. OD by lysate and supernatant of PC-3 cells compared to PC-3 cells (as positive control) and cell-free media (as negative control) following 3 hours of incubation with 0.4 mg/mL MTT. 20,000 cells were seeded per well of 96-well plates and were allowed to grow in phenol red-free FCS-containing RPMI for 24 h. The media was then replaced with fresh media of the same type with or without FCS. Two hours later, cells supernatant and lysates were isolated, and all samples had MTT added. Data shown as mean OD of triplicate wells and error bars represent SD (standard deviation). *** *p*-value < 0.0001.

We questioned if the active viable cells’ secretome or the released intracellular contents from dead/lysed cells might also contribute to MTT reduction and formation of formazan crystals in the extracellular space. To address this question, we performed the MTT assay on the lysate and supernatant of PC-3 cells (20,000 cells seeded per well of a 96-well plate) cultured in phenol red-free RPMI with or without fetal calf serum (FCS). The corresponding cell-free culture media and non-lysed PC-3 cells cultured with the same density were, respectively, used as negative and positive control samples. As shown in Figure 7, we did not observe any significant difference in the OD levels between PC-3 cells’ lysate, supernatant, and the corresponding culture media samples, while the OD levels by the non-lysed PC-3 cells were significantly higher than the corresponding cell-free samples.

Furthermore, using phase-contrast microscopy with magnifications of 4× and 20×, we did not observe any formazan crystals in the wells containing cell lysate, cell supernatant, and culture media after 3 h of incubation with MTT, while needle-shaped extracellular crystals, as well as intracellular formazan granules, were observed in the cell-containing wells.

This data shows that MTT reduction is not mediated by the enzymes released from PC-3 cells as a result of active secretion or cell lysis using a RIPA buffer or at least, it is not at a significant level detectable by the microplate reader or light microscopy. Even though, it should be considered that lysis buffers can denature proteins. Therefore, cell lysis by a RIPA buffer might not accurately mimic other mechanisms of loss of cell membrane integrity and release of intracellular contents. Pulsed-sonication is another method of lysing cells that might cause less denaturation of proteins. This method was used by Liu et al. [5] to lyse erythrocytes and B12 (glial) cells and assess MTT reduction by cell lysate. They proposed that the lysate of these cells reduces MTT, reporting a very low OD value of 0.081 for erythrocyte lysate and a range of OD values for B12 cell lysate (0.509 to 1.226 for different protein concentrations) measured at 570–630 nm. However, it was not mentioned if (or how) the reported OD values have been normalized. It has not either been clarified if any of the cell lysate samples correspond to the same number of cells used in measuring the OD of non-lysed cells. In addition, it has not been assessed if the OD differences between the different concentrations of cell lysate and non-lysed cells were simply a result of optical differences or indicate different levels of MTT reduction. Nevertheless, the data of the two studies are not comparable due to the differences in the studied cell types, the cell lysis method, and the range of wavelength at which the OD was measured.

The possible contribution of the released intracellular content to MTT reduction is of more importance when the tested condition or treatment causes cells to lose membrane integrity and release a considerable amount of intracellular biomolecules. This can also be a potential concern raised from the presence of reactive oxygen species in the secretome of radio/chemo-induced growth-arrested giant cells (SASP, mentioned earlier in Section 2.1.1) that may contribute to MTT reduction [13,35,37]. This possibility should be considered and investigated to prevent biased measurements of cell viability based on OD values.

#### 2.1.5. Washing Cells’ Supernatant Following MTT Reduction

The fact that we did not observe extracellular needle-shaped formazan crystals in cell lysate, cell supernatant, and cell-free culture media samples suggests that the observed needle-shaped crystals in the cell-containing wells were not a result of extracellular abiotic reduction of MTT, but their origin is intracellular. This is in agreement with previously published studies where the appearance of needle-shaped formazan crystals was associated with the gradual decrease in the intracellular granular form of purple formazan [41] and even continued for up to 3 h after washing out the remaining MTT from the cells’ supernatant [5]. Therefore, despite controversies on the mechanism of formation of extracellular formazan, it appears most likely that they are of intracellular origin in our case, and therefore ought to be considered in the final measurement in the MTT assay.

**Figure 8:**
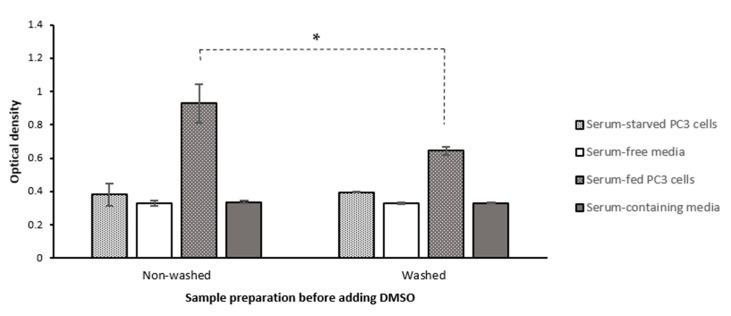
Washing cells after incubation with MTT removes extracellular formazan crystals: The effect of washing the cells’ supernatant on the OD levels. 20,000 PC-3 cells were seeded per well of 96-well plates and were allowed to grow in phenol red-free RPMI with or without FCS for 26 h. The cells were then incubated with 0.4 mg/mL MTT for 3 h. Wells were then divided into two groups: (i) Non-washed wells: 85 µL of supernatant in each well was removed before adding DMSO so that 25 µL was left in each well. (ii) Washed wells: Supernatant was totally removed. Then wells were washed two times with PBS and 25 µL of fresh media was added before adding DMSO. Data shown as mean OD of triplicate wells and error bars represent standard deviation. * One-tail *t*-test *p*-value = 0.025.

This is however commonly overlooked in performing MTT assays when cells are washed before adding the formazan-solubilizing reagent. Extracellular formazan crystals have even been assumed as a source of false positivity in cell viability measurements [2]. The washing step has also been justified as a way to remove the remaining non-reduced MTT in the supernatant. This does not seem to be necessary because firstly, adding surfactant would stop the process of MTT reduction by disrupting the integrity of the cells, and secondly, MTT cannot interfere with the OD measurements as we later show in this study. We, therefore, questioned if the washing step could introduce a significant error in the MTT assay measurements by excluding, at least a part of, intracellularly formed extracellular formazan crystals. To answer this question, we compared the MTT assay results on PC-3 cells with and without performing the washing step. As shown in Figure 8, our results showed that removing the supernatant and washing cells before adding DMSO caused a significant decrease in the OD levels (*p*-value = 0. 02) in serum-fed cells (using a one-tail T-test). However, washing the cells did not significantly affect the OD in cells that were serum-starved for 26 h before MTT addition (Figure 8, *p*-value = 0.7).

This result indicates that the washing step could act as a confounder variable when there is a considerable amount of extracellular formazan (as in serum-fed cells). Therefore, washing the supernatant could result in eliminating a significant proportion of intracellularly formed extracellular formazan from the OD measurements. However, as the level of MTT reduction and extracellular formazan was extremely low in severe serum starvation, the washing step did not significantly affect the OD measurements. This shows that the confounder effect of washing depends on the level of MTT reduction and subsequent extrusion of crystals into the supernatant. In other words, the level of formazan that washing excludes from OD measurements is not consistent and controllable between different samples and could result in misleading data. Hence, our data suggests it is preferable not to wash the cells.

**Figure 9:**
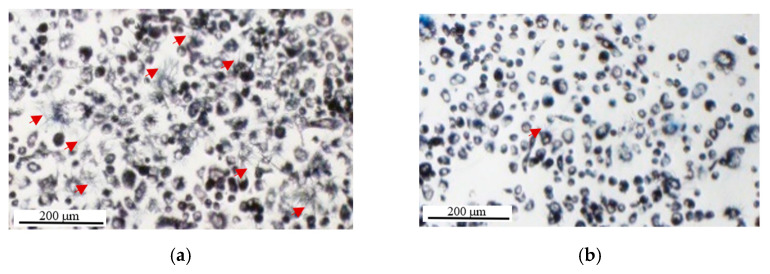
Washing cells following MTT incubation removes intracellularly formed extracellular formazan. Light microscopy images (×4) showing the effect of washing the cells’ supernatant before solubilizing formazan on removing formazan crystals. 20,000 PC-3 cells were seeded per well of 96-well plates and were allowed to grow in phenol red-free RPMI with FCS for 26 h. The cells were then incubated with 0.4 mg/mL MTT for 3 h. The wells were then divided into 2 groups: (**a**) Non-washed wells, (**b**) Washed wells in which the supernatant was totally removed, and then cells were washed two times with PBS and fresh media (with the same volume as non-washed wells) was added to each well. Red arrows show the extracellular needle-shaped formazan crystals which are much fewer than the non-washed sample.

It should be noted that in our experiment we only assessed the effect of washing, and not just removing the supernatant. Based on light microscopic observations (Figure 9) we could observe that needle-shaped crystals were suspended in the supernatant, and we therefore expect that removing the supernatant will also reduce OD. Comparing the mean OD of cells normalized to the corresponding cell-free media in two separate experiments also showed that media removal before adding DMSO (even with no washing step) can cause a significant decrease in the OD levels (Figure S3). Furthermore, as shown in Figure 9, the washing step also appears to cause a decrease in the cell density probably as a result of removing loosely-attached cells by washing the supernatant. Therefore, the washing step can cause the removal of a portion of both intracellular and extracellular formazan that are both products of intracellular MTT reduction. Although the scale of this effect is required to be tested, it seems removing/washing the supernatant is a potentially error-producing extra step for which there is no convincing justification as previously suggested [45]. Removing or washing the cells’ supernatant before adding formazan-solubilizing solvents (such as DMSO), therefore, should be avoided to include the effect of both intracellular and extracellular formazan into the light absorption measurements.

#### 2.1.6. Culture Media

The components present in culture media could potentially affect the MTT assay measurements. This could conceivably arise due to optical interference such as light absorbance or scattering, chemical reactions such as abiotic reduction of MTT, and biological effects on the cells’ viability, growth, and metabolic activity which could consequently affect the total level of MTT reduction. To investigate such effects, we measured the UV-Vis absorption spectrum as well as the OD levels of different compositions of the cell-free culture media with and without MTT. We also compared the results of the MTT assay on PC-3 cells grown in different compositions of culture media.

##### Optical Interference

It has been previously shown that experimental conditions could affect the produced formazan UV-Vis spectrum and cause a shift in its peak absorbance wavelength [18]. Hence, to make the OD measurements a more accurate representation of formazan concentration, it is ideal to optimize the measurement wavelength for each experimental condition. On the other hand, using a standard measurement window is also important for the sake of data comparisons. In this study, we measured the OD at 570/10 nm which is roughly around the peak absorbance wavelength reported for formazan [18] and is the most commonly applied measurement window for the MTT assay [43,45,46,47].

All the components present in the microplate wells could potentially affect optical density if they scatter or absorb light at the same wavelength as the assay measurement window. We, therefore, assessed if culture media including RPMI, phenol red, FCS, as well as the MTT reagent itself could interfere with OD measurements.

- RPMI and MTT

**Figure 10:**
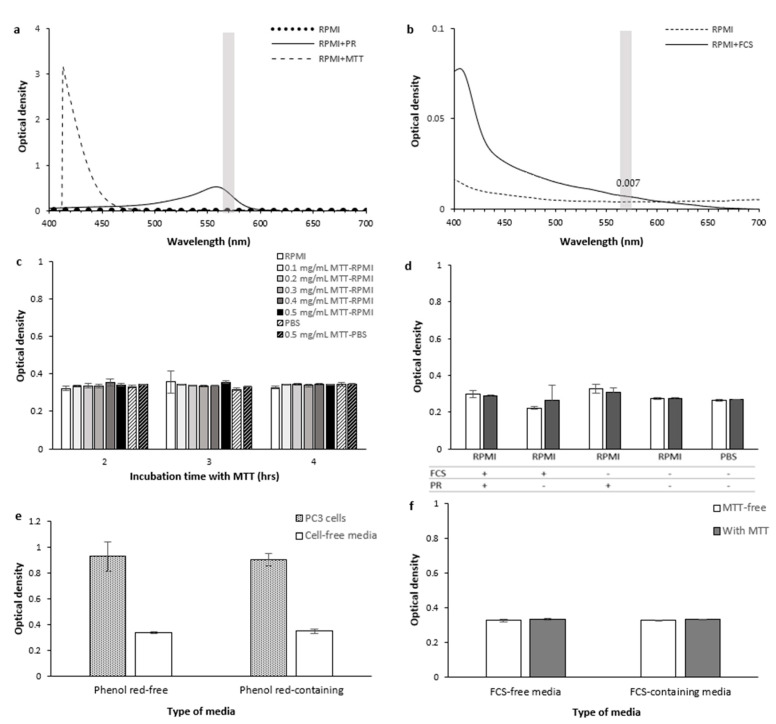
Optical and chemical interference by culture media components. (**a**) UV-Vis absorbance spectrum of RPMI with and without phenol red (PR) or 0.4 mg/mL MTT, (**b**) UV-Vis absorbance spectrum of RPMI without and with 10% fetal calf serum (FCS). Grey regions indicate the analysis window for OD measurements. Measurements in UV-Vis (**a**,**b**) were made against PBS for background, (**c**) OD of cell-free RPMI/PBS incubated with different concentrations of MTT for 2 to 4 h, (**d**) OD of cell-free RPMI with and without PR and/or FCS (10%) incubated with MTT-free PBS or 0.4 mg/mL MTT–PBS solution for 3 h before OD measurements in the MTT assay, (**e**) Effect of phenol red on MTT assay results on PC-3 cells. 20,000 PC-3 cells were seeded per well of 96-well microplate and allowed to grow for 26 h before 3 h of incubation with 0.4 mg/mL MTT. Corresponding cell-free media were used as negative control, (**f**) The OD of RPMI with and without FCS, incubated with MTT-free PBS or 0.4 mg/mL MTT –PBS solution for 3 h before OD measurements. Data shown as mean OD of triplicate wells and error bars represent standard deviation.

As shown in Figure 10a, the UV-Vis spectroscopy showed that RPMI with or without MTT did not show a notable absorbance at 570/10 nm. The peak absorbance of MTT-RPMI solution at shorter wavelengths is consistent with the MTT–DMSO solution having a peak at 410 nm as shown previously [18].

As shown in Figure 10c, the OD level of RPMI (containing phenol red and 10% FCS) is similar to the PBS OD level. In addition, absence or presence of different concentrations of MTT within the culture media or PBS did not notably affect the OD levels.

Taken together, these data suggest that RPMI itself as well as the presence of MTT in the cells’ supernatant do not cause an optical interference with MTT assay measurements. Therefore, there is no need to remove the remaining MTT before solubilizing formazan as we mentioned earlier.

- Phenol red

As shown in Figure 10a, the presence of phenol red in RPMI caused an absorption peak around 560 nm which falls within the assay measurement window. Microplate reader measurements showed that the scale of the effect of adding phenol red to cell-free media of the same type on the OD levels depended on the presence or absence of FCS and MTT in the media (Figure 10d). When we cultured PC-3 cells in the FCS-containing RPMI, the presence of phenol red in the culture media did not cause a significant change in the OD levels (*p*-value > 0.05, Figure 10e) as suggested in previous studies [3,45,48]. This can be due to the change that occurs in the color of phenol red to yellow as a result of acid production by cells. Therefore, the absorbance spectrum of phenol red would alter in the presence of cells and may not anymore interfere with the assay measurements. However, this color change may not be consistent between different cell types, culture conditions, and/or tested treatments. Regardless, the presence of phenol red is not essential for incubating cells with MTT. We, therefore, suggest using phenol red-free media before incubating cells with MTT to minimize the measurement errors as a result of any possible interference from phenol red.

- FCS

The UV-Vis absorbance peak of RPMI containing 10% FCS at around 410 nm did not overlap the assay measurement window (570/10 nm). However, the presence of 10% FCS in RPMI caused a negligible rise in the RPMI OD levels (0.007) within the assay window (Figure 10b). As for phenol red, the microplate reader measurements showed that adding FCS to cell-free media of the same type resulted in various effects on the OD levels depending on the presence or absence of the phenol red and MTT in the media (Figure 10d,f). Long-term serum starvation could affect the metabolic behaviour of cells and the level of MTT reduction. However, as we show later, the effect of starving cells from serum for a short time appears to be negligible on the assay measurements. Hence, we recommend to grow cells in serum-containing media and replace the media with serum-free media before MTT addition to minimise any error in measurements. Using a single serum batch for each experiment is also recommended to avoid inconsistencies in growth conditions between tested samples.

##### Chemical Interference

Culture media components can potentially act as a confounder in the assay measurements not only through direct optical interference but also through unforeseen chemical reactions with MTT such as reducing MTT or catalyzing MTT reduction. Such reactions can result in the production of new chemicals, such as formazan, that can change the measured optical density. We earlier showed MTT itself does not optically interfere with the assay measurement. Therefore, observing a difference in OD as a result of MTT addition to the media could be an indicator of a chemical reaction between MTT and the media components such as abiotic reduction of MTT by the media components. Hence, we compared the OD levels between cell-free media with and without MTT to assess potential chemical interference by culture media components.

As shown in Figure 10d,f, the presence or absence of MTT in RPMI did not significantly affect the OD levels regardless of the presence or absence of FCS and/or phenol red (*p*-value > 0.1). The OD levels of culture media (RPMI containing phenol red and 10% FCS) incubated with varying concentrations of MTT solution (0 to 0.5 mg/mL) for 2 to 4 h before adding DMSO were also similar (Figure 10c). These results suggest that no remarkable abiotic reduction of MTT was mediated by RPMI, FCS, and/or phenol red for at least up to 4 h of incubation. This is consistent with data from a previous study suggesting that RPMI does not contribute to MTT reduction [49]. A similar result was reported for RPMI up to 6 h of MTT incubation when measuring OD at 490 nm [49]. The effect of MTT concentration on the OD levels of FCS-containing RPMI (5% FCS) was also shown to be small with up to 6 h of MTT incubation [43]. The same study however reported an increase in the enhancing effect of MTT concentration on the OD values from 17 to 24 h of MTT incubation. Notably, MTT incubation in all the samples in this study was followed by 24 h of SDS incubation [43] which means the actual MTT incubation lasted even longer than the mentioned times. As formazan absorbance is typically measured at or before 4 h of MTT incubation, the probable chemical effect of media at longer time points would not be an issue in the assay measurements. The MTT reduction by other types of culture media was also proposed including photocatalytic reduction of MTT by DMEM (Dulbecco’s Modified Eagle Medium) [42] and MTT reduction by M199 due to the presence of reducing agents such as ascorbic acid and retinol in the media [49].

Abiotic reduction of MTT by human serum albumin (HSA) was also suggested by Funk et al. [50], where they observed an increase in OD with increasing HSA concentration. However, they did not exclude the optical interference of HSA, and OD was measured at 540 nm rather than 570 nm [50] so their data remain inconclusive. Variability between batches of FCS may also cause diversity in chemical interference of serum in MTT assay measurements.

Not only is it possible that culture media and serum directly reduce MTT, but they may also influence MTT reduction that is mediated by tested chemicals such as plant extracts [51] and flavonoids [47,52]. Such influence has been attributed to the presence of components such as glucose, vitamins, and enzymes in the culture media [47,52]. Other forms of chemical interference by culture media components with MTT assay measurements have been also reported such as the reaction of EMEM (Eagle’s Minimum Essential Medium) with a formazan–DMSO solution [48] and the interaction of serum proteins with some tested drugs [53]. This further highlights the importance of considering and testing the potential interferential effect of culture media in MTT assay measurements, data analysis, and data interpretation.

##### Biological Effects

Culture media provides nutrients for cell growth, proliferation, and metabolism. Consequently, the composition of culture media, such as the presence or absence of serum, could influence the MTT assay measurements by influencing the cells’ biological behaviour such as the level of metabolic activity and thus MTT reduction. To investigate how the effect of serum starvation on the metabolic activity of cells would be reflected in the MTT assay measurements, we compared the MTT assay results between cells grown in serum-free and serum-containing media. We also aimed to investigate the effect of the commonly applied serum-free conditions on the assay measurements.

To assess the effect of short-term starvation, we seeded PC-3 cells in phenol red-free serum-containing RPMI and allowed them to grow for 24 h. Then, we changed the media with serum-free or serum-containing (10% FCS) media and incubated the cells for a further 2 h before the addition of MTT. Corresponding cell-free media were used as negative controls. We also imaged these cells from 2 h before changing the media up to 3 h after MTT addition using live phase-contrast imaging microscopy (Incucyte ZOOM).

In a separate experiment, we investigated the effect of long-term starvation on PC-3 cells to assess the effect of extreme nutritional stress on the MTT assay results. We seeded PC-3 cells in phenol red-free RPMI with or without 10% FCS and allowed them to grow for 26 h before MTT addition. We also imaged these PC-3 cells after 3 h of incubation with MTT using light microscopy.

**Figure 11:**
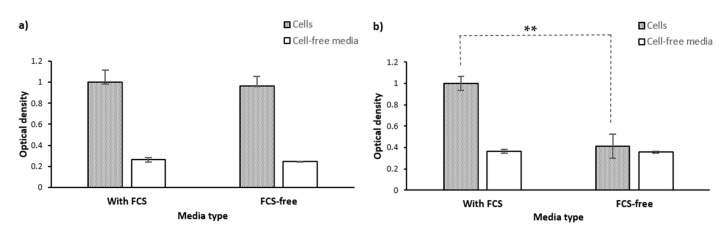
Serum starvation can affect MTT reduction. 20,000 PC-3 cells were seeded per well of 96-well plates and grown in phenol red-free RPMI with 10% FCS for 24 h. The media was then replaced with serum-free or serum-containing (10%) RPMI and cells were incubated for 2 h (**a**) or 26 h (**b**). Cells were then incubated with 0.4 mg/mL MTT for 3 h before OD measurements. Data shown as normalized mean OD of triplicate wells and error bars represent standard deviation. ** *p*-value = 0.005.

As shown in Figure 11a, serum-fed cells’ OD (normalised to 1.0) was slightly higher than short-term serum-deprived cells (0.96), but not significantly (*p*-value = 0.5). Contrarily, serum starvation of cells for 26 h caused a decrease in OD (from 1.0 to 0.41 OD) compared to cells grown with FCS (Figure 11b). No difference was observed over the same time if cells were not present (both 0.35–0.36 OD). Therefore, the difference in OD between serum-fed cells and cells starved of serum for 26 h was most likely due to a difference in intracellular MTT reduction and formazan production, and not optical or chemical interference of FCS. The underlying mechanism could be attributed to the effect of the presence of serum in the culture media on the cells’ nutritional state, intracellular signaling, metabolic activity/pathways, and the level of oxidative stress, cell cycle, cell viability, and cell proliferation [54,55,56,57]. The effect from two hours of serum starvation was not at a level to cause a significant change in the OD level.

**Figure 12:**
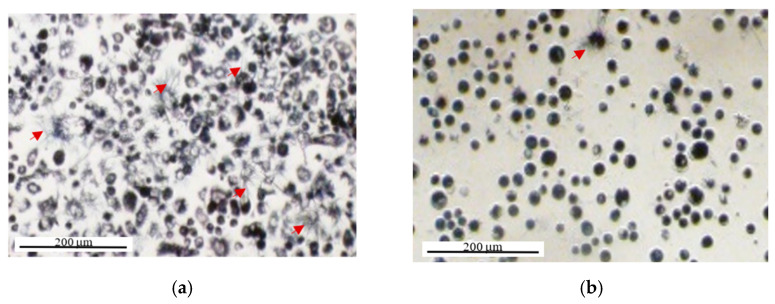
Extremely serum-starved cells show apoptotic morphologic features, yet still reduce MTT to formazan. Light microscopy images (×4) comparing cell morphology and formazan formation in serum-fed (**a**) and 26-h serum-starved cells (**b**) as per Figure 11. The purple color of cells is a result of formazan aggregation inside the cells. Red arrows show the extracellular needle-shaped formazan crystals which are much fewer in serum-starved cells.

As shown by light microscopy (Figure 12) the majority of cells that were serum-starved for 26 h showed a round morphology (some with blebbing) compared to the normal spindle shape of the majority of serum-fed cells. While the spindle shape of PC-3 cells could be indicative of a viable status and adherence, the round shape indicates cell detachment and can be a morphological indicator of cell apoptosis [58,59,60,61]. However, cell death is a staged process usually taking a few hours to complete [59] and cells with a round shape could be at different stages of cell death, where at earlier stages, they still show some level of metabolic and enzymatic activity and could reduce MTT as shown by formazan formation in these cells (Figure 12) which is consistent with previous studies [62]. Therefore, while it could be that all cells are undergoing apoptosis, there is still metabolic activity that could erroneously be interpreted as representing viable cells. Here, however, we observe that most cells appear to be undergoing apoptosis with a 26-h starvation and that the OD measurements appear consistent with this observation (Figure 9b).

Contrary to the long-term serum starvation, the morphology of the majority of short-term serum-deprived cells seemed to be normal as for serum-fed cells. This is consistent with the OD measurements showing no significant difference in OD between serum-fed and short-term serum-deprived cells and suggests that 2 h of serum starvation does not affect the biological status of the cells (i.e., cell viability and metabolic activity) at a significant level. This further confirms that biological activity, rather than optical/chemical effects of FCS, impacted the assay measurements. This also highlights the capability of the MTT assay to reveal major differences in viability between the two cell populations. However, the contribution of round cells in the earlier stages of apoptosis to MTT reduction could confound the quantitative measurement of cell viability using the MTT assay. The cell death process provides a spectrum of viable to non-viable and apoptotic cells rather than a binary living versus dead status. Therefore, morphological studies along with the MTT assay could give more accurate information on the viability percentage of a cell population. Complementary assays on cell viability or metabolic activity could also be considered to provide more robust data. 

The response of cells to serum starvation is a diverse, dynamic, and complicated process and depends on different factors such as incubation time, experimental conditions, and cell type [56]. This could explain the inconsistency in reports on the effect of serum starvation on MTT assay measurements. For instance, Twentyman et al. reported higher OD values in serum-starved cells compared to serum-fed cells [48]. On the other hand, the variability in the composition between batches of serum in the culture media could also result in diverse biological responses. Therefore, eliminating serum has been proposed to lead to greater homogeneity and synchronicity between different cells/cell populations [56].

Taken together, these observations further show the necessity of optimization experiments before performing the MTT assay for different experimental conditions, cell types, and types of culture media.

#### 2.1.7. Tested Treatment (Gold Nanoparticles)

Many nanoparticles are well known to give rise to distinctively colored solutions. To assess possible optical interference from red-color Au-PEG NP solutions on the MTT assay measurements, we measured the UV-Vis absorption spectrum of 0.2 nM Au-PEG NPs in the phenol red-free serum-free RPMI. We also measured the OD of the phenol red-free RPMI incubated with a concentration series of Au-NPs (0, 0.05, 0.5, and 5 nM) with and without MTT to investigate possible optical and chemical interference in the MTT assay. We then compared MTT assay measurements on PC-3 cells containing different NP concentrations. We also did the same comparison on the lysate and supernatant of these cells.

**Figure 13:**
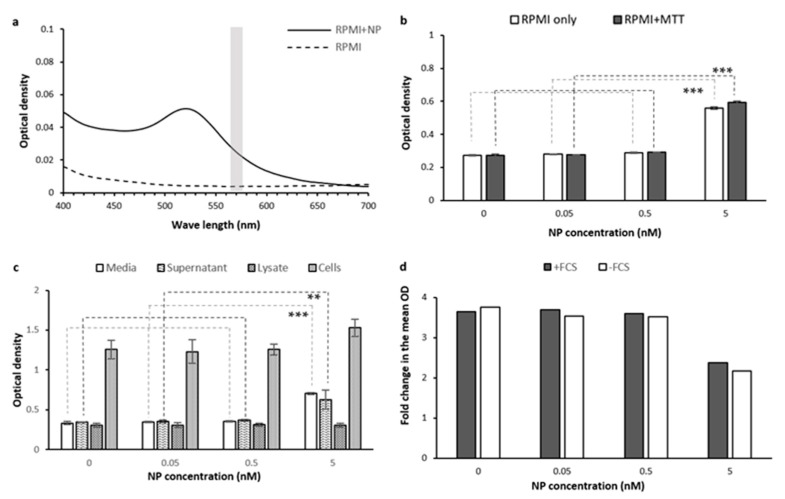
Au-PEG NPs can interfere with MTT assay measurements. (**a**) UV-Vis absorption spectrum of RPMI with and without 0.2 nM Au-PEG NPs. Grey regions indicate the analysis window for OD measurements in the MTT assay. Measurements in UV-Vis were made against PBS for background, (**b**) OD of cell-free phenol red-free FCS-free RPMI containing different concentrations of Au-PEG NPs after 3 h of incubation with MTT-free PBS or 0.4 mg/mL of MTT–PBS solution, (**c**) OD of PC-3 cells treated with Au-PEG NPs compared to their lysate, supernatant, and cell-free culture media. 20,000 PC-3 cells were seeded per well of 96-well plates in phenol red-free RPMI with 10% FCS for 24 h. Media was then replaced with serum-free RPMI containing different concentrations of Au-PEG NPs and cells were incubated with NPs for 2 h. The supernatant of some cells was then removed and loaded in separate wells for MTT incubation. The remaining cells were lysed using the RIPA (Radioimmunoprecipitation assay) lysis buffer and covered with the same volume of NP-free media. The wells containing cells, cell lysate, cell supernatant, and cell-free culture media were then incubated with 0.4 mg/mL MTT for 3 h before OD measurements, (**d**) The fold change in OD levels for PC-3 cells in Figure 13c compared to their corresponding cell-free culture media with the same concentrations of Au-PEG NPs. The same data is presented as dark bars for cells incubated with the same concentrations of Au-PEG NPs in serum-containing media (RPMI + 10% FCS). Data shown as mean OD of triplicate wells and error bars represent SD (standard deviation). ** *p*-value < 0.005, *** *p*-value < 0.0001.

Figure 13a shows the UV-Vis spectra of NP-containing media exhibiting an absorption peak in the range of ~500–600 nm with the highest absorption at 520 nm. Adding the NPs to the media up to a concentration of 0.05 nM did not cause a significant change in OD (Figure 13b). However, the highest concentration of 5 nM resulted in a significantly greater OD (*p*-value < 0.0001) indicating interference of Au-NPs at 5 nM.

There are some NPs known to catalyze redox chemistry at their solid–liquid interface and could reduce MTT [34,42]. To assess if our Au-PEG NPs have a similar effect, we compared the OD levels between the media solutions of the same Au-NP concentrations with and without MTT (Figure 13b). We observed a small, but significant increase in OD from 0.54 to 0.59 when adding MTT to the 5 nM Au-PEG NP solution (*p*-value = 0.003). In lower concentrations of NPs though, adding MTT did not cause a difference in OD. This could suggest a minor catalytic effect on MTT reduction or abiotic reduction of MTT that is mediated by Au-PEG NPs in our case.

The MTT assay on PC-3 cells cultured in phenol red-free serum-free RPMI also showed a non-significant increase in OD (from 1.25 to 1.53) when comparing 0.5 nM to 5 nM NP concentration (Figure 13c). On the other hand, significant increases in OD were measured for the cells’ supernatant and culture media containing 5 nM NPs (Figure 13c, *p*-value < 0.005 for cell supernatant and <0.0001 for culture media). The OD of cell lysate after treatment with different NP concentrations (0, 0.05, 0.5, and 5 nM) were very similar (~0.3 OD, Figure 13c). In this case, the greatest influence of NPs on OD measurements was due to NPs in the supernatant. While it is not uncommon to skip the washing step when evaluating the cytotoxic effect of gold nanoparticles [63,64,65,66], we found that washing is necessary to remove this interference. If NP uptake is much greater, such as at longer time points or concentrations, we would also expect that NPs within the cells themselves will also interfere with OD measurements. In this case, an artificially high OD measurement could erroneously suggest greater biocompatibility.

The NPs could potentially affect the intracellular reduction of MTT if they interfere with cellular biochemistry. To investigate such a possibility, we first needed to exclude the optical confounding effect of Au-NPs on the OD. In this regard, we calculated the fold change in OD in the presence of cells, compared to cell-free media containing the same NP concentrations (Figure 13d). The results showed small differences in the fold change between non-treated and treated cells with 0.05 and 0.5 nM NP concentrations. However, the fold change decreased from 3.5–3.7 times to 2.1–2.3 times when the Au-NP concentration increased to 5 nM (Figure 13d). This could indicate that 2 h of incubating PC-3 cells with 5 nM of Au-PEG NPs decreases the MTT reduction by cells.

Measuring the cytotoxic effect of AuNPs using the commonly used approach for inferring cell viability or IC50 (half maximal inhibitory concentration), based on the MTT assay results [63,64,65,66,67,68,69,70,71,72], however, would erroneously show an increase in cell viability when cells were treated with 5 nM NPs. For instance, calculating cell viability percentage based on the OD measured for PC-3 cells grown in FCS-free phenol red-free RPMI (Figure 13c) would show around a 22% increase in cell viability ([treated cell OD (1.53)/non-treated cell OD (1.257)] × 100 ≈ 121.72%). By considering the confounding effect of Au-NPs on OD (Figure 13d), MTT reduction in cells treated with 5 nM Au-NP have actually decreased around 60% compared to non-treated cells, [fold change in OD for treated cells ((2.165)/fold change in OD for non-treated cells (3.763)] × 100 = 57.53%). This shows the importance of using cell-free samples as negative controls to exclude the potential confounding effect of Au-NPs, or any other tested treatments, to prevent erroneous measurements. Nevertheless, it has been a common practice to overlook this effect in measuring cytotoxicity or biocompatibility of Au-NPs [63,64,67,68,69,70,71], including PEG-coated or PEG-containing gold NPs/nanocomposites [65,66,72], for diagnostic or therapeutic purposes. While we have shown the confounding effect of Au-NPs on MTT assay measurements, such effects need to be considered for any other kind of treatments, as shown for TiO_2_ nanoparticles [34,42], silicon nanowires [73], flavonoids [47,52], and plant extracts [51].

### 2.2. MTT Reduction Measurement at the Single-Cell Level Using Image Cytometry

Image cytometry has been used in a few studies to measure intracellular formazan production at the single-cell level, called MTT Image cytometry [14,42,62]. The measurement has been done either by measuring the intracellular light intensity in inverted grayscale images taken by bright-field microscopy [14] or by calculating the light absorbance using optical filters within the formazan absorption spectrum wavelength range (around 500–570 nm) [42,62].

Single-cell cytometry is generally a valuable tool for assessing cell population heterogeneity [74], identifying subpopulations involved in macroscale observations [75], and modeling the stochastic nature of cell biology [76]. The MTT Image cytometry can also address the confounding effect that some non-cellular variables (such as interference from culture media) have on the bulk MTT assay measurements. Moreover, single-cell cytometry could address some pitfalls of the MTT bulk assay by assessment of changes in the cell morphology along with the single-cell level MTT reduction. This was for instance previously shown by detecting cisplatin-induced growth-arrested giant cells known as dormant cancer cells that can reduce MTT 10 times more than non-treated cells and cause an underestimation of the proliferation block caused by cisplatin in the bulk measurements [35]. However, MTT Image cytometry may still have some of the limitations of the bulk MTT assay such as the optical interference from treatments uptaken by cells (e.g., Au-PEG nanoparticles) or MTT reduction by apoptotic cells. Moreover, the probable presence of intracellularly formed extracellular crystals and the confounder effect of excluding them from single-cell level measurements should not be overlooked. The MTT Image cytometry studies, however, have been limited to the analysis of intracellular formazan [42,62].

**Figure 14:**
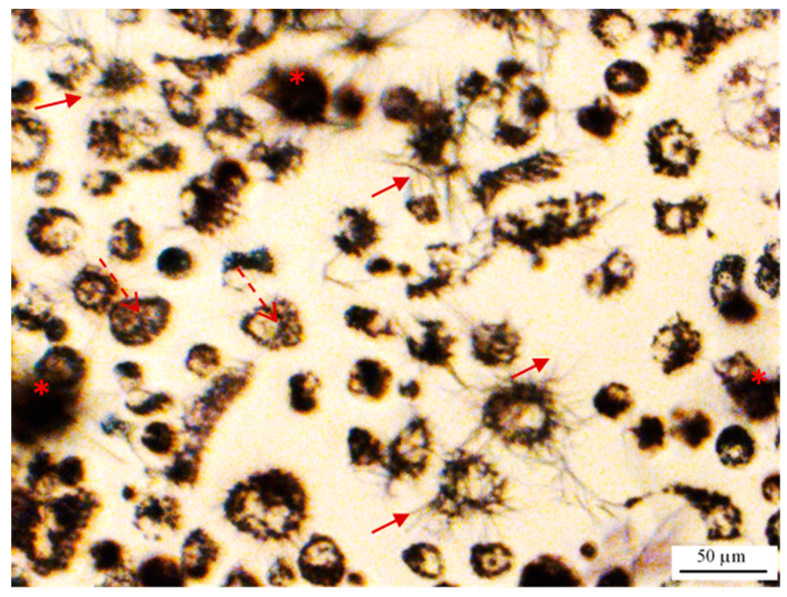
The heterogeneous location and morphology of intracellularly formed MTT-derived formazan. Light microscopy image (×20) showing formazan aggregates in PC-3 cells incubated with 0.4 mg/mL MTT for 3 h. Purple formazan aggregates are granular inside the cells (dashed arrows), but needle-shaped when located on the cell surface (red arrows), or suspended in the supernatant (stars).

Imaging PC-3 cells after 3 h of MTT incubation using light microscopy, we observed a considerable number of extracellular formazan crystals (Figure 11a and Figure 13). While some of the crystals appeared to be attached to the cell surface, others appeared to be suspended in the supernatant as they were observable at a different focal plane from the attached cells (Figure 14). In a separate experiment using the Incucyte live-cell imaging system (phase-contrast microscopy, ×10 magnification); however, we observed many fewer extracellular crystals at around the same incubation time (Figure S4). This could be explained by the Incucyte system using a focal plane fixed at the bottom of the wells. Therefore, the imaging system capabilities should also be considered in interpreting the data in MTT Image cytometry.

While the common assumption in light microscopy imaging studies has been that the needle-shaped crystals remain attached to the cell surface [5,40,41], our observations indicate that they can detach from the cell surface over time and suspend in the supernatant. Therefore, using light microscopy, it is difficult to associate any observed crystal aggregate with a specific cell. To include all the intracellularly formed formazan in the measurements, the imaging time needs to fall before the extrusion of intracellular formazan for every single cell. Imaging all the single cells also needs to be done at the same time point after MTT addition, when a sufficient level of MTT has been reduced to enable comparative analysis between single cells and evaluating intercellular heterogeneity. Imaging PC-3 cells from 4 h before up to 3 h after MTT addition, using the Incucyte live imaging system (×10 magnification), we observed that the first needle-shaped extracellular formazan crystals appear at ~2.5 h after MTT addition (Figure S4)—that was close to our measurement time point for the bulk assay. However, the appearance of extracellular formazan crystals at the cell surface has been reported to be very time-dependent and vary dramatically across different cell lines as well as cells of the same population [5,40,41]. While in some studies, no or a few extracellular crystals were observed after 1.5–4 h of MTT incubation [14], in other studies, the crystals have been reported to appear on the surface of 2–10% of cells as soon as 30 min after MTT addition [5,41], the majority of cells between 2 [41] and 3 h [5] after incubation, and 100% of the cells after 3–6 h of incubation with MTT [5,40]. The gradual appearance of these extracellular crystals was associated with a gradual decrease in intracellular formazan-containing granules that started to form as soon as 5 min after MTT addition [5,40,41]. These time variations could be attributed to the dependence of the formazan extrusion rate on the cell type [5,77], the cell growth phase (slower in exponential phase compared to stationary phase) [5], and the tested treatments [40]. Hence, determining an optimum imaging time point generalizable to all or at least the majority of cells can be difficult.

## 3. Materials and Methods

### 3.1. Defining the Optimum Cell Density, MTT Concentration, and MTT Incubation Time in the Bulk MTT Assay on PC-3 Cells

To assess the effect of cell density, MTT concentration, and MTT incubation time on the MTT assay results and find the optimal values for these parameters, we seeded PC-3 cells (ATCC, Manassas, VA, USA) of passage number 12 on three 96-well Microplates (Corning^®^, New York, NY, USA) with an area per well of0.32 cm^2^, varying the cell seeding density (0, 5000, 10,000, and 20,000 cells per well, respectively, corresponding to an estimated 0%, 22.5%, 45%, and 90% of confluency for PC-3 cells at the time of the OD measurements (27–30 h after seeding) based on our light microscopy observations) and allowed them to grow in a humidified incubator at 37 °C with 5% CO_2_ for 24 h (the doubling time of PC-3 cells) in phenol red (PR)-containing RPMI (Gibco Roswell Park Memorial Institute 1640, Thermo Fisher Scientific, Waltham, MA, USA), containing 10% fetal cow serum (Gibco™ Fetal Bovine Serum, Thermo Fisher Scientific, Waltham, MA, USA) and 1% penicillin–streptomycin (Gibco™ Penicillin-Streptomycin, Thermo Fisher Scientific, Waltham, MA, USA), of 100 µL per well in total. Although no nanoparticle was added to the cells at this stage, to make the conditions consistent with our later experiments with Au-NPs, we considered the required time of 2 h for incubating the cells with Au-NPs, so MTT reagent (Invitrogen™, Thermo Fisher Scientific, Waltham, MA, USA) was added to cells 26 h after seeding. We added 10 µL of a concentration series (0, 1, 2, 3, 4, and 5 mg/mL) of MTT solution in PBS to each well. Each condition (considering both MTT concentration and cell density) was repeated in triplicates in each 96-well plate. We also loaded 6 wells per plate with 100 µL of PBS- as negative controls to 3 of which we added 10 µL of PBS (no MTT), and to the other 3 we added 10 µL of 5 mg/mL MTT–PBS solution. Each of the 3 plates was used to assess the MTT reduction and formazan crystal formation at a specific time point of 2, 3, and 4 h after MTT addition. At each time point, 50 µL of DMSO (Sigma-Aldrich, Burlington, MA, USA) was added to each well of the according plate following removing 85 µL of the supernatant. The DMSO was then mixed with the cell–MTT suspension for 10 s using a plate shaker followed by incubating the mixture at 37° C for 10 min to allow DMSO to solubilize the formed crystals making a homogenous solution. The 570/10 nm wavelength light absorption in each well was then measured in the OD (optical density) unit using the FlUOstar Optima Microplate Reader (BMG LABTECH, Ortenberg, Germany).

### 3.2. The Effect of Au-PEG NPs and Culture Media Components In-Solution

To assess the possible interference of the used cell culture media and polyethylene glycol (PEG)-coated gold nanoparticles (Au-NPs) in the light absorption measurement via light absorption or abiotic reduction of MTT by the media components, we first measured the UV-Vis absorption spectrum of RPMI, with or without FCS, PR, and/or PEG-coated Au-NPs (PEG-Au-NP). The PBS with or without PEG-Au-NP samples were used as negative controls. The PEG-Au-NP solution in Milli Q water (see the description of synthesis [78] and characterization of the nanoparticles in the Supplementary Materials) was added to RPMI solutions or PBS to make the final NP concentration of 0.2 nM, while the same volume of Milli Q water with no NPs was added to the corresponding RPMI solutions as NP-free negative controls. The volume of 2250 µL of each solution with or without NPs was loaded per well of two 12-well plates in duplicates and incubated at 37 °C for 2 h. The volume of 225 µL of MTT–PBS solution or just PBS was then added to each of the duplicates and incubated at 37 °C for a further 3 h. The UV-Vis absorption spectrum was then measured using the Evolution™ 300 UV-Vis Spectrophotometer (Thermo Fisher Scientific, Waltham, MA, USA). The PBS with no MTT was used as the blank sample.

We also measured the 570/10 nm wavelength light absorption by the same solutions without and with 3 different dilutions of Au-PEG-NPs (0.05, 0.5, and 5 nM). The volume of 100 µL of each solution was loaded in each well of two 96-well plates. Each condition, considering the presence or absence of FCS or PR in media as well as the NP concentration, was repeated in triplicates in each plate. After 2 h of incubation of the solutions at 37 °C, 10 µL of 4 mg/mL MTT solution in PBS or just PBS was added to each well of respectively the first and the second plates. The plates were then incubated at 37 °C for further 3 h. The 570/10 nm wavelength light absorption in each well was then measured in OD (optical density) unit using the FlUOstar Optima Microplate Reader (BMG LABTECH, Ortenberg, Germany).

### 3.3. The Effect of Supernatant Washing, Serum Starvation, and Phenol Red in Cells and In-Solution

Based on our results in experiment 1, the optimum values of the cell seeding density, stock MTT concentration, and cell–MTT incubation time for performing the MTT assay on PC-3 cells were shown to be, respectively, 20,000 cells/well of a 96-well plate, 0.4 mg/mL, and 3 h.

To assess the validity of the MTT assay in the assessment of the viability of PC-3 cells, we evaluated the effect of serum starvation on the MTT assay results. We also aimed to evaluate the effect of the presence of PR in the culture media in both serum-fed and serum-starving cells. In this regard, we seeded 20,000 PC-3 cells/well in two 96-well plates using 100 µL/well of PR-free or PR-containing RPMI (containing 1% Penicillin–Streptomycin) and containing none or 10% FCS as the culture media. The same volume of the same types of culture media was loaded in wells with no cells as corresponding negative controls. Each condition was repeated in one (considering the presence of PR) or two (considering the presence of cells and/or FCS) series of triplicates in each plate. The cells were allowed to grow in a humidified incubator at 37 °C with 5% CO_2_ for 24 h. Twenty-six hours after seeding, 10 µL of 4 mg/mL MTT solution in PBS or just PBS were added to each well, and the plates were incubated at 37 °C for a further 3 h. After 3 h of incubation, in the first triplicate series of each condition as well as in PR-containing wells, we removed 85 µL of the supernatant from each well and added 50 µL DMSO per well, mixing following the same protocol in the previous experiment.

As in many studies and MTT routine protocols, all or a volume of supernatant is removed and/or cells are washed immediately before adding DMSO; therefore we questioned if removing the media and washing the cells could change the measured light absorption by washing out some of the extracellular formazan crystals and if this decrease in absorption level would be consistent between samples or act as a confounder variable. To address this question, in the second triplicate series of each condition (considering the presence of cells and/or FCS), we washed each well twice with 100 µL of PBS and then added 25 or 110 µL of fresh media of the same type to each well of respectively the first and the second plate. In the first plate, DMSO was added as for the first series of wells, and the 570/10 nm wavelength light absorption was then measured in each well using the FlUOstar Optima Microplate Reader (BMG LABTECH, Ortenberg, Germany). The second plate was used for imaging using the light microscopy mode of the Olympus IX83 Fluorescence microscope, so no DMSO was added to the second plate since DMSO destroys the cells and solubilizes formazan crystals. For non-washed wells in the first plate, 85 µL of media was removed before adding DMSO. In the second plate that was used for imaging, however, no media was removed in non-washed wells.

### 3.4. The Effect of Au-PEG NPs, Cell Lysate, and Cell Secretome in Cells

To assess if the released intracellular enzymes following cell lysis or the viable cells’ secretome could also contribute to MTT reduction and the formation of formazan crystals, we performed the MTT assay, respectively, on the PC-3 cells’ lysate and supernatant. We seeded PC-3 cells in PR-free FCS-containing RPMI (including 1% penicillin–streptomycin) in two 96-well plates. The cells were allowed to grow for 24 h in a humidified incubator at 37 °C with 5% CO_2_ equipped with the Incucyte^®^ ZOOM Live-Cell Analysis System (Sartorius AG, Göttingen, Germany), where imaging of one triplicate series of cell-containing wells in one of the plates was planned every 10 min starting from 2 h before NP treatment up to 3 h after MTT addition. Imaging was performed in phase-contrast mode with ×10 magnification. Twenty-four hours after seeding, the cells’ supernatant was removed, and cells were washed twice with PBS. A volume of 100 µL of FCS-free or FCS-containing RPMI containing different concentrations of Au-PEG-NPs-water solutions (0, 0.05, 0.5, 5 nM) was then added to each well. Each condition (considering the NP concentration and the presence of FCS in the media) was repeated in 2 series of triplicates. The same solutions were also loaded in empty wells with no cells (100 µL/well) as negative controls in triplicates.

After 2 h of incubation with NPs in the same incubator, the first series of the cells’ supernatant in one of the plates was removed and loaded in corresponding wells of a separate empty plate one by one. Then the cells were washed once with PBS and were lysed by adding 20 µL of RIPA buffer (containing no protease inhibitor) to each well followed by a mechanical scratch using the 20 µL pipette tip. Then 80 µL of fresh media of the same type, but with no NP was added to each well to make the total volume consistent with the volume in other wells. The cells in the second series of wells, that were imaged by the Incucyte ZOOM, were left intact as positive controls. Then 10 µL of 4 mg/mL MTT–PBS solution was added to every well and the plates were incubated in the previously used incubator at 37 °C for 3 h. After 3 h of MTT incubation, images were taken from all the plates using the same live-cell analysis system. Then, without removing any supernatant, 150 µL of DMSO was added to each well. The DMSO was then mixed with the wells’ contents and the mixtures were incubated at 37° C for 10 min to allow DMSO to solubilize the formed crystals. The 570/10 nm wavelength light absorption was then measured in each well using the FlUOstar Optima Microplate Reader (BMG LABTECH, Ortenberg, Germany).

## 4. Conclusions

In this study, we aimed to investigate the validity, reliability, and limitations of the MTT assay at cell population (bulk assay) and single-cell (image cytometry) levels. We also aimed to unfold some aspects of the underlying mechanism behind the MTT assay and how these aspects need to be considered in designing and performing MTT assay experiments, as well as analyzing and interpreting the assay results.

Cell number, MTT concentration, and MTT incubation time effect assay measurements. Our results demonstrate the necessity to optimize these parameters for each cell line/experimental condition. The optimum values of these parameters are achieved when the assay could optimally reveal the differences between different cell populations by allowing cells to show their maximal capability in MTT reduction but do not cause a significant level of cell death/toxicity before the assay measurements. Furthermore, to compare the effect of different treatments/conditions on MTT reduction, we recommend to do the comparative analysis changing these parameters to avoid misleading comparisons and have a broader perspective on the treatment response.

It is in such an optimum condition where the OD values could be applied as an approximate estimation of the average level of MTT reduction in each cell population. However, the OD level is not a simple representation of just one parameter such as cell viability, cell proliferation, or metabolic activity, but is a sum of many factors at the single-cell and cell population level as well as other cellular factors such as cell growth phase and the rate of MTT uptake and formazan extrusion that all could be potentially affected by the tested treatment(s)/culture media. This complexity could even cause false-positive results when some treatments are being tested using MTT assay. For instance, radiation is known to cause cell death through DNA damage, but it increases cell membrane permeability [73] and mitochondria number [74] and activity [32] which both could enhance formazan formation through increasing MTT uptake and reduction, respectively.

To be able to relate the differences in OD measurements to changes in a specific variable such as cell viability, we need to make sure of the consistency of other effective variables among the conditions. The correlation pattern between the assessed dependent variable and the OD values for each cell type/experimental condition also needs to be assessed and referenced in the data analysis.

Other than the cellular factors affecting OD measurements, there are also potential non-cellular confounders that need to be considered. These factors include the optical and/or chemical interference of culture media components, tested treatment(s), and formazan-solubilizing agents. Chemical interference includes abiotic extracellular reduction of MTT and/or other types of chemical reactions that could indirectly affect the OD measurements.

The aim of our study was to identify potential confounding variables that may affect the assay measurements. Therefore, all the measurements in this study are used as a tool to represent potential pitfalls, limitations, and misinterpretations of the assay and the necessity of optimization experiments. To gain a quantitative measure of the influence of each of these variables, technical replication would be required and may be necessary depending on the system being studied and for what purpose. Considering our model system, our data indicate the optimum values for performing bulk MTT assays to be 3 h of incubation of 20,000 cells/100 µL/well in a 96-well plate (seeding density) with 10 µL of 4 mg/mL MTT–PBS solution.

In our experiments, we did not observe any significant optical or chemical interference by MTT or RPMI itself. Our data did not show any significant effect of the presence of phenol red in the culture media on the MTT assay measurements on PC-3 cells. However, presence of phenol red and 10% FCS caused some changes in the OD levels of media solutions depending on the media composition. Therefore, we recommend replacing media with phenol red-free serum-free media before MTT addition to minimize any possible error. Our results showed that long-term serum starvation significantly affects cell viability (based on assessing cell morphology) and thus alters the MTT assay bulk measurements. However, short-term serum starvation did not cause a significant change in OD measurements. Our data also suggests that enzymes released by PC-3 cells did not significantly contribute to extracellular MTT reduction and thus was not a confounder variable in our MTT assay measurements. However, this may vary between different cell types, experimental conditions, and treatments.

The Au-PEG NPs at 5 nM concentration led to significant optical interference. This confounding effect could be minimized by removing the supernatant and washing the cells before incubation with MTT or using proper cell-free controls with the same NP concentration.

We showed that the origin of extracellular needle-shaped formazan crystals in our experimental conditions is most likely intracellular and thus these crystals need to be included in MTT assay measurements. This means that washing or removing the cells’ supernatant before DMSO treatment could result in biased measurements by excluding intracellularly formed extracellular crystals as well as loosely-attached cells that have contributed to MTT reduction.

While the direct confounding effect of some of the extracellular and cell population-level variables (such as cell number/proliferation) could be addressed in image cytometry by measuring only the intracellular absorbance/intensity along with cellular morphologic assessments at the single-cell level, there are still some limitations and considerations to use this approach: First, the extremely heterogeneous rate of formazan extrusion makes the reliability of such a measurement questionable. Image cytometry, however, may still be a valuable tool for specific cell types in which the rate of formazan extrusion is sufficiently slow as to not cause bias in intracellular measurements. Second, observing formazan granules inside the cells at different stages of cell death is another limitation of image cytometry which could be addressed by considering cell morphological features along with formazan intensity in the data interpretation. Third, the possible optical interference of the tested treatment(s) in the intracellular absorption measurements also needs to be considered and tested.

To summarize, as a tool to measure the cell viability, metabolic activity of cells, and/or treatment cytotoxicity, the MTT assay necessitates several considerations (Table 1). These may be addressed by performing extra optimization experiments which could be a time-consuming and tedious, yet important, process. In many cases, complementary assays are recommended to assist in the interpretation of MTT assay measurements. Nevertheless, the assay has been commonly used as the main basis of such measurements, overlooking the limitations and the necessity of performing optimization assays.

## Figures and Tables

**Table 1:** Considerations in performing MTT assay.

Does the MTT assay represent what you aim to measure? Have you optimized the following parameters? ▪ cell seeding number and density ▪ MTT concentration ▪ MTT incubation time Do your culture conditions (such as culture media type, presence of serum, and phenol red in the media, etc.) affect your assay measurement by either optical or chemical interference? Are any effects of tested treatments considered that could affect the final OD measurements in a direct or indirect way? ▪ MTT uptake and/or extrusion (e.g., cell membrane permeability/integrity) ▪ Cell number (e.g., proliferation) ▪ Cell metabolism (e.g., chemo/radio-induced senescence-like phenotype) ▪ Cell secretome (e.g., chemo/radio-induced senescence-associated secretory phenotype) ▪ Background absorbance and scattering ▪ Abiotic reduction of MTT
If your aim is a quantitative measurement of cell viability, have you defined how OD values relate to the number of cells?
Based on your answers to the above questions: ▪ Is MTT assay an appropriate tool to answer your research question? ▪ Have you considered using appropriate control samples to reduce risk of misinterpretation of data? ▪ Have you considered complementary assays to confirm MTT assay results?

## Data Availability

The data that support the findings of this study are available from the corresponding author upon reasonable request.

## Supplementary Materials

The following are available online at www.mdpi.com/article/10.3390/ijms222312827/s1.
