# Supplementary material for: The MTT Assay: Utility, Limitations, Pitfalls, and Interpretation in Bulk and Single-Cell Analysis
Source: Int J Mol Sci. 2021 Nov 26;22(23):12827. doi: 10.3390/ijms222312827 (PMC8657538; doi:10.3390/ijms222312827)
Supplement: Supplementary file 1 [file ijms-22-12827-s001.zip › ijms-1443072-supplementary.pdf]

Supplementary Materials

Research Article

The MTT Assay: Utility, Limitations, Pitfalls, and Interpretation in Bulk and Single-Cell Analysis

Mahshid Ghasemi, Tyron Turnbull, Sonia Sebastian and Ivan Kempson *

University of South Australia, Future Industries Institute, Adelaide, SA, Australia
* Correspondence: ivan.kempson@unisa.edu.au

Supplementary Materials and Methods

Nanoparticle synthesis

To synthesize the Au-PEG nanoparticles, we prepared the gold seed solution using the Turkevich method [1]. In brief, we added 1 mL of 1% $AuCl_4$ solution (Sigma Aldrich, CAS ID: 16961-25-4) to 100 mL of Milli Q water and heated it until it boiled. We then added 5 mL of trisodium citrate solution (Sigma Aldrich, CAS ID: 6132-04-3, 30 mg/mL) to the boiling solution. The reaction was continued for 10 minutes after which we removed the solution from heat and left it to cool down.

To prepare the Au-PEG nanoparticles, we added 62 μL of short-PEG (S-PEG, Sigma Aldrich, CAS ID: 866889-02-3), 338 μL of long-PEG (L-PEG, Sigma Aldrich, CAS ID: 165729-81-7), and 400 μL of Milli Q water to 1 mL of the gold seed solution. We sonicated the mixture for 10 minutes at room temperature and then refrigerated it overnight. After 12 hours, we centrifuged the solution and resuspended the pellet in fresh Milli Q water to make the final Au-PEG solution.

Nanoparticle characterization

We used transmission electron microscopy (TEM) to image the nanoparticles (Figure S1), and dynamic light scattering measurements (DLS) to confirm the particle size and conjugation of PEG to the gold nanoparticle (Figure S2). Based on the TEM imaging and DLS measurement, the gold seed size ranged between 15 to 20 nm. The hydrodynamic size of Au-PEG nanoparticles ranged between 20 to 30 nm based on DLS measurements.

Supplementary Figures

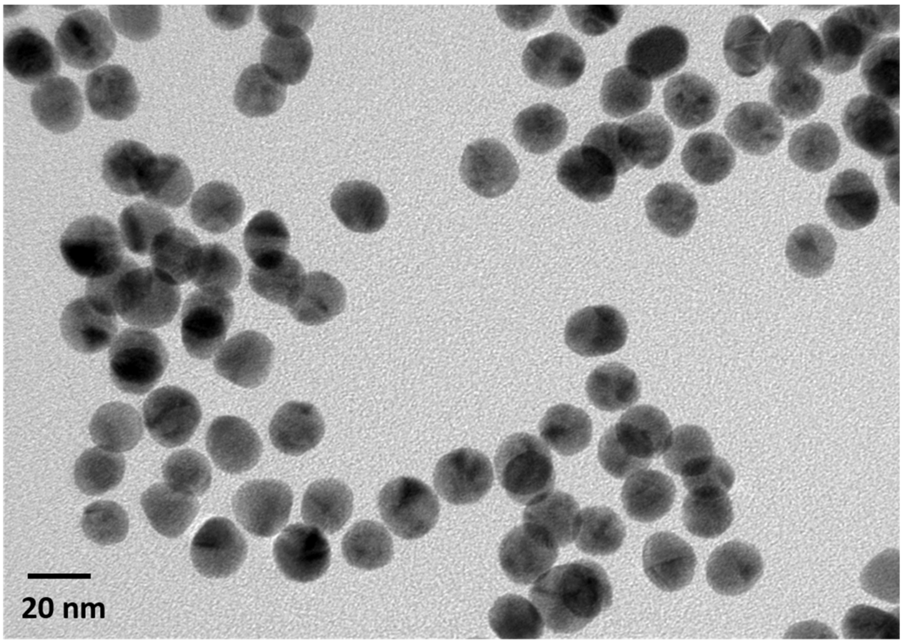

Figure S1. Transmission electron micrograph of gold nanoparticles

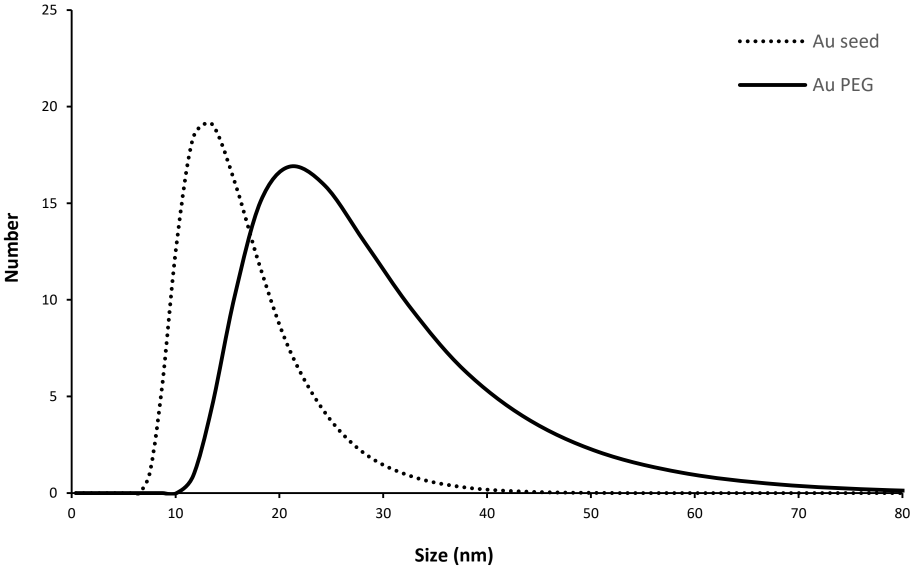

Figure S2. Dynamic light scattering measurements of Au seed and Au-PEG nanoparticles

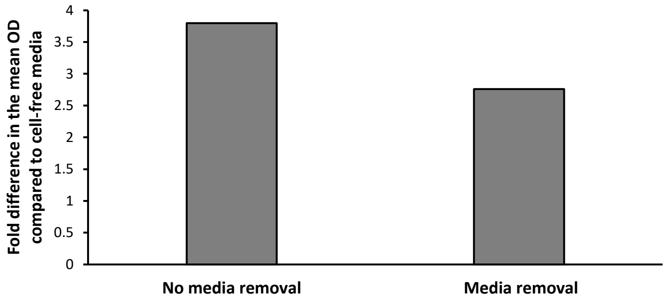

Figure S3. Media removal before adding DMSO decreases the measured OD levels. 20,000 PC-3 cells were seeded per well of 96-well plates and grown in phenol red-free RPMI with 10% FCS for 26 hours. Cells were then incubated with 0.4 mg/mL MTT for 3 hours. For the "no media removal" samples, 150 µl of DMSO was added to each well without any media removal. For the "media removal" samples in a separate experiment, 85% of media was removed before adding 50 µl DMSO to each well. Cell-free samples were used as controls. Data shown as the fold change in the mean OD of triplicate wells compared to the corresponding cell-free media samples in the same experiment.

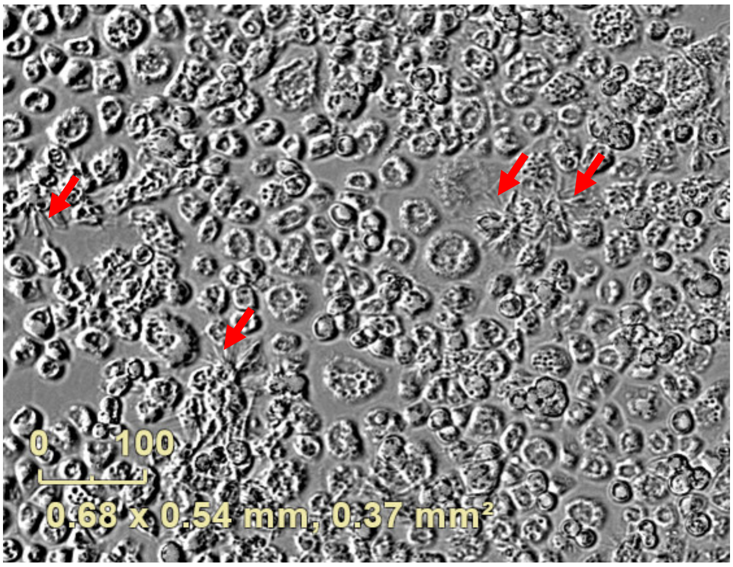

Figure S4. **Needle-shaped formazan crystals.** The Incucyte phase-contrast live cell image (×10) showing the first extracellular needle-shaped formazan crystals (red arrows) that appeared after 2.5 hours of incubating PC-3 cells with 0.4 mg/mL MTT.

Reference

1. Turkevich, J.; Stevenson, P.C.; Hillier, J. A study of the nucleation and growth processes in the synthesis of colloidal gold. *Discuss. Faraday Soc.* **1951**, *11*, 55–75.
